# Deweyan Reflex Arc: The Origins of an Idea

**DOI:** 10.3389/fpsyg.2022.829387

**Published:** 2022-03-24

**Authors:** Da Dong, Wei Chen

**Affiliations:** ^1^Center for Brain, Mind, and Education, Shaoxing University, Shaoxing, China; ^2^Department of Psychology, Shaoxing University, Shaoxing, China; ^3^Interdisciplinary Center for Philosophy and Cognitive Sciences, Renmin University of China, Beijing, China

**Keywords:** John Dewey, cognitive science, reflex arc, organicism, functional psychology

## Introduction

Tracing the origins of an idea is an important work. Historian Bloch (1953) emphasized that usually, scrutinizing the origins of an idea is the beginning of the explanation of the idea. Besides, it should be noted that an origin of a scientific or metaphysical idea is not the same as an entirely scientific or metaphysical explanation. John Dewey's 1896 article “The Reflex Arc Concept in Psychology,” which was published in the nineteenth century, is still frequently used in currents of cognitive sciences (to name a few, e.g., Chemero, 2009; Anderson, 2014; Clark, 2016; Gallagher, 2017). This is an unusual case. Here, we term Dewey's idea of reflex arc as “Deweyan reflex arc.” In 1896, as it has been shown, Dewey's refinement of the concept of reflex arc did not just abandon the elementalist version of reflex arc; but rather, he embraced a radically different version of which latterly dubbed “functional psychology” (cf. Brett, 1921; Boring, 1950; Murphy and Kovach, 1972).

Although the Deweyan reflex arc enjoys a high reputation in the history of psychology, it is far from clear that this kind of reinterpretation is one of the best candidates for counting as a genuine definition of “reflex arc.” The 1896 article did not follow the orthodox operational definition of “reflex arc” in the nineteenth century (Müller, 1838). Dewey did not even present neuroanatomical achievements in spinal reflex at that time (Dewey, 1896). Until now, a reflex arc commonly has five distinct components: receptor, sensory neuron, interneuron, motor neuron, and effector. In current neuroscience textbooks, for example, Luo (2021) asserts that specific cognitive functions of neural systems are ultimately based on diverse neuronal circuits and their compositions. Spinal reflex arc circuits represent the simple case of circuits; and knee-jerk reflex (only including a sensory neuron and a motor neuron) is the simplest type of reflex (Luo, 2021, pp. 15–17). Kandel et al. (2021) conclude the principles of neuronal circuits as: each neuronal cell is one part of a circuit; cellular signaling pathways are organized in the same way in all neuronal cells; the reflex circuit is a starting point for understanding neural mechanisms (Kandel et al., 2021, p. 56). Briefly, we find that the current orthodox definition of “reflex arc” is still consistent with Deweyan reflex arc's opponent, elementalist reflex arc. Elementalists (representatives include Wilhelm Wundt) literally held that each mental phenomenon or process could be broken down into its underlying smallest units or composite elements. The founder of structural psychology, Edward Titchener, embraced this doctrine and even went farther: he almost asserted that psychologists' only task was to specify determinate mental elements (Titchener, 1898, 1899). In this case, many philosophers would not agree that Dewey provided an accurate explanation of reflex arc *per se*.

In this opinion piece, we aim to provide a short historical survey on the soundness of Dewey's idea as one kind of “reflex arc.” The 1896 article, in one sense, could be seen as the culmination of Dewey's early period of “New Psychology” from the year 1884 (Dewey, 1884, 1887, 1930). Given this background, we first contrast key differences between the Deweyan reflex arc and the elementalist reflex arc. Then, we present that those two competing ideas can be separately traced back to two radically different origins of *reflex* in the seventeenth century: Thomas Willis's organicism and René Descartes' mechanism.

## Manuscript Formatting

### In the Period of “New Psychology”

In the period of “New Psychology,” Dewey's thoughts “drifted away” from Neo-Hegelianism to pragmatism or so-called “instrumentalism” (Dewey, 1930, p. 20). This idea, to a large extent, grew out of Dewey's early intimate connection with Neo-Hegelianism. Recall Dewey's reminiscence: “there were, however, also ‘subjective' reasons for the appeal that Hegel's thought made to me; it supplied a demand for unification…” (Dewey, 1930, p. 19; for a review of Hegelian organicism see e.g., Bubbio, 2017). However, it is not an exaggeration to say that the Deweyan reflex arc and Dewey's other early ideas are immersed in a unification of explanation. Dewey insisted on unifying all phenomena of nature, thereby including mind-body relationships.

Doubtlessly, Dewey directly opposed neuro-reductionism and neuro-centrism. In the 1884 article “The New Psychology,” he did not think that the mind can be reduced to the brain either ontologically or methodologically. Dewey claimed that a real discipline of mental science must be independently established on the facts (and conditions) and methodology of the psychological domain (Dewey, 1884). In an article entitled “Soul and Body,” he even implicitly invoked an idea of “spinal soul” (i.e., even the spine has its own mind), contrasting with the *cerebral soul*. Dewey argued: the mental is “homogeneously” related to the physical. Units of nervous system, the neurons, no matter where they are located, at the cerebral, the spinal, or the peripheral, are of the same kind and are of equal importance (Dewey, 1886, p. 242). In a word, the mind cannot belong to one proper part of the body; it is “immanent” in the body (Dewey, 1886). Furthermore, Dewey seemed to not believe in localization of mental functions, see: “…we see the entire failure of all attempts definitely to localize the higher intellectual functions. …any attempt to find a sharply marked I out centre must be forever in vain.” (sic) (Dewey, 1886, p. 258). Localization of reflex action in the spinal cord, and of speech action in the cerebrum, in Dewey's own words, “is a difference of degree, not of kind.” (Dewey, 1886, p. 255). Dewey motived a functionally organizing principle of brain and cognition. The Deweyan reflex arc is one of the outcomes.

### Some key Differences Between Deweyan arc and Elementalist Reflex arc

The 1896 article is acclaimed as the official manifesto of functional psychology, and Dewey is recognized as the founding father of Chicago school (Boring, 1950, p. 554; Li, 2020, p. 120). However, to be clear, involvement was not what Dewey had intended. He stayed away from the debate over functional versus structural psychology. Ironically, the name “functional psychology” was probably not created by Dewey himself or any one advocate but by a rival, Titchener. It is Titchener (1898) who made a distinction between two possible approaches of consciousness studies: structural psychology studies what consciousness *is*, while functional psychology studies what consciousness is *for*; the latter shall be seen as a discipline of applied psychology. Titchener noticed the 1896 article and claimed that it contained an idea of functional psychology (Titchener, 1898, pp. 451–452). Then, Titchener (1899) elaborated further that structural psychology shall be more fundamental than functional psychology; the latter must be established on the basis of the former. Afterward, it was the younger psychologist Angell (1907) who officially founded the Chicago school. The facts: Dewey never made a response to Titchener's attacks and barely used the title of “functional psychology.” Throughout his life, Dewey kept a distance from psychological movements and debates. The idea of sensorimotor coordination or perception-action cycle underlying the Deweyan reflex arc is greatly ahead of experimental achievements of that time.

Four key differences between the Deweyan and elementalist reflex arcs (including Titchener's functional psychology) are provided (see Table 1). First, note that the idea of the Deweyan reflex arc is not a product of Dewey's neuroanatomical studies on decapitated animals (nineteenth century model organisms of spinal reflex studies, e.g., spinal frog). Depending on observed objects, the Deweyan reflex arc radically departs from the dominant “decapitated” definition of “reflex arc.” In other words, the elementalist reflex arc is appropriate for dead animals, while the Deweyan reflex arc, in the other approach, is all about living phenomena. Second, it is fair to say that reflex unity in Deweyan view is continuous, while in elementalist view it is discontinuous; see: “the fact is that stimulus and response are not distinctions of existence, but teleological distinctions, that is, distinctions of function” (Dewey, 1896, p. 365). Third, in the Deweyan view, the brain, as the highest level of neural systems, is indispensable for a reflex arc (see Dewey's experimental paradigm, the child-candle problem) (James, 1890, p. 25; Li, 2020, pp. 121–122), while the “decapitated” elementalist reflex arc is functionally isolated from the brain, so to speak. Lastly, the Deweyan reflex arc, in some sense, pioneered organicist criticisms of reflex arc in the early twentieth century (Nichols, 2009). Goldstein (1995) and Merleau-Ponty (1967), etc., intended to radically shift the scientific paradigm of brain and cognition studies against the background of Russian and Soviet reflexology or American behaviorism. In the opposite sense, the elementalist view could be classified as mechanical philosophy.

**Table 1:** Four issues on which the Deweyan reflex arc and the elementalist reflex arc differ.

**Issue**	**Deweyan** **Reflex Arc**	**Elementalist** **Reflex Arc**
Are model organisms alive or dead?	Alive	Dead
Continuous or discontinuous unity?	Continuous	Discontinuous
Is the brain an indispensable part?	Yes	No
Philosophical tradition	Organicism	Mechanism

### Tracing the Two Origins of “Reflex” in the Seventeenth Century

In this section, we claim that the Deweyan and elementalist reflex arc could be separately traced back to two radically different origins of “reflex.” The problem of reflex used to be a big challenge and, to some extent, culminated in Charles Sherrington's (1906) integrative reflex theory of the spinal cord. Owing to Sherrington, the perplexing idea of spinal soul “had been put to rest” (Bennett and Hacker, 2003, p. 42). Generally, the elementalist reflex arc could be traced back to Descartes' *De Homine* (1662), while the Deweyan reflex arc, in our view, could be traced to Willis's *Cerebri Anatome* (*The Anatomy of the Brain*) (1664). Descartes belongs to mechanistic materialism, while Willis's philosophy is closely akin to an early tradition of organicism (cf. Foster, 1901; Gault, 1904). In Sherrington (1906), Canguilhem's (1994) and other scholars' views, Willis rather than Descartes is the real originator of the idea of *reflex*, “the thing, the word, and the notion” (Canguilhem, 1994, p. 188). However, the origins of *reflex* might be left unsettled.

Descartes actually did not use the word and the idea of reflex; however the orthodox view was finally re-molded in a Cartesian mechanical way. In a well-known illustration of the 1662 book, one can see a big baby whose foot at first gets close to a pile of fire unintentionally and then withdraws the foot automatically in a very short time to avoid injury (Descartes, 1998, p. 118). Descartes described there are animal spirits in the cavities of the brain that pass through fibers of tubes of the nerves; animal spirits arrive at all the muscles of the body, of which related with foot “pull the foot away from the fire,” and of which related with other muscles “make the hands move and the whole body turn” (Descartes, 1998, pp. 117–119). Metaphorically, Descartes regarded bodily movements as the workings of “artificial fountain.”

In contrast, to Willis, the word “reflex,” in the very beginning, literally meant *reflection* of lights. The brain was regarded as the one and single illuminant; in one sense, both stimulus and response are all homogeneous *reflected* “lights.” Canguilhem (1994) accused that the mechanist Descartes merely focused on the hydraulic automechanism of involuntary muscular movements, and that he ignored the *reflected* relationship of the sensory and the motor; see: “the Cartesian theory is […] certainly mechanical, but it is not the theory of the reflex” (Canguilhem, 1994, p. 184). In contrast, with the aid of the analogy of “light,” Willis “conceived of the anatomical structure of the nervous system as radiant rather than ramified, with the brain emitting nerves as the sun emits rays” (Canguilhem, 1994, p. 187). Thus, the first use of the descriptive word *reflexion* by Willis should be seen as an analogy of “light.” Indeed, “it is from this word that we get the word “*reflex*” (Finger, 2000, p. 91). Hence, metaphorically, Willis saw the same thing as the workings of “illumination,” *reflection* of light (Willis, 1681, 1683).

Based on discussions made throughout the article (especially the four key points summarized in Table 1), we claim that the Deweyan reflex arc might be traced to the real originator of “reflex,” Willis. In our opinion, Dewey not only gave a novel perspective but also provided one kind of genuine explanation of reflex arc *per se*.

## Conclusion

Lakoff and Johnson (1999) praised Dewey as one of the two greatest philosophers of embodied cognition (the other figure is Maurice Merleau-Ponty). In the realm of 4E cognition (embodied, enactive, embedded, and extended), scholars scrutinize the Deweyan reflex arc in different aspects (cf., e.g., Venturelli, 2012; Vaesen, 2014; Chirimuuta, 2020). Clark (2016) reinterprets the Deweyan reflex arc in the current of predictive processing; the ongoing Deweyan reflex arc cycle now is remolded by Bayesian modeling. Now, cognitive scientists' favor of the Deweyan reflex arc is overtly spreading to the burgeoning area of predictive processing (cf. Friston, 2016; Metzinger and Wiese, 2017).

Our proposal has limited scope. Tracing the origins of the Deweyan reflex arc is not the same as explaining this idea. The former question is suitably classified in the realm of the history of ideas or intellectual history. However, given the limited space, much more historical investigations are needed to strength this subject matter.

## Author Contributions

Both authors listed have made a substantial, direct, and intellectual contribution to the work and approved it for publication.

## Funding

This study was supported by the National Social Science Fund of China (Grant No: 20CZX015).

## Conflict of Interest

The authors declare that the research was conducted in the absence of any commercial or financial relationships that could be construed as a potential conflict of interest.

## Publisher's Note

All claims expressed in this article are solely those of the authors and do not necessarily represent those of their affiliated organizations, or those of the publisher, the editors and the reviewers. Any product that may be evaluated in this article, or claim that may be made by its manufacturer, is not guaranteed or endorsed by the publisher.
